# Supplementary material for: Effects of CALR-Mutant Type and Burden on the Phenotype of Myeloproliferative Neoplasms
Source: Diagnostics (Basel). 2022 Oct 23;12(11):2570. doi: 10.3390/diagnostics12112570 (PMC9689478; doi:10.3390/diagnostics12112570)

Supplementary Figure S1. (A) Scatter plots between *CALR*-mutant burden and white blood cell (WBC) count and absolute neutrophil count (ANC) in MPN patients with type 1-like *CALR* mutation and **(B)** scatter plots between *CALR*-mutant burden and WBC count and hemoglobin (Hb) level in MPN patients with type 2-like *CALR* mutation. Circles of the same color in each group indicate the same patient.

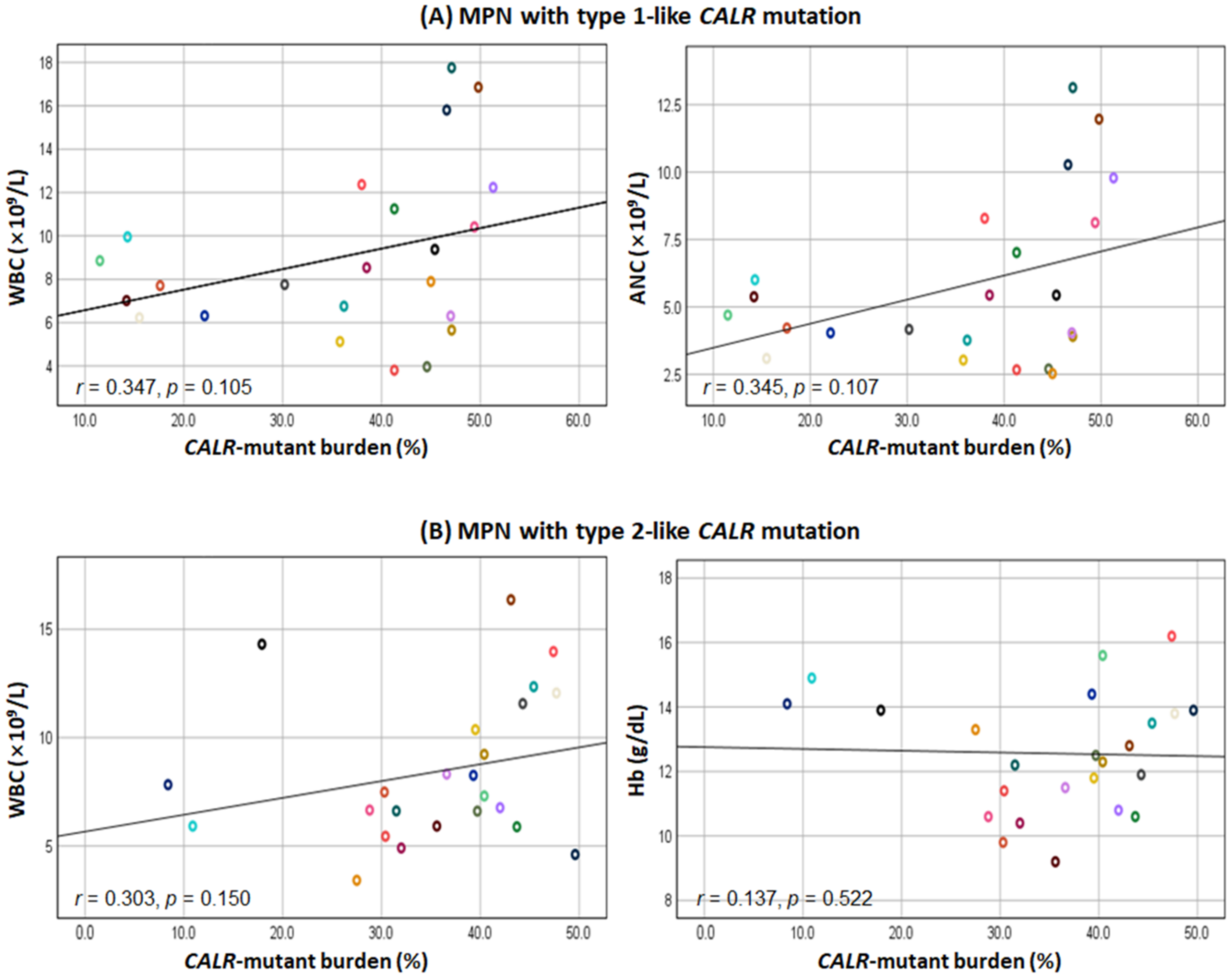

Supplementary Figure S2. Scatter plots between *CALR*-mutant burden and white blood cell (WBC) count, absolute neutrophil count (ANC), hemoglobin (Hb) level and platelet count in patients with **(A)** *CALR*-mutated ET and **(B)** *CALR*-mutated PMF. Circles of the same color in each group indicate the same patient.

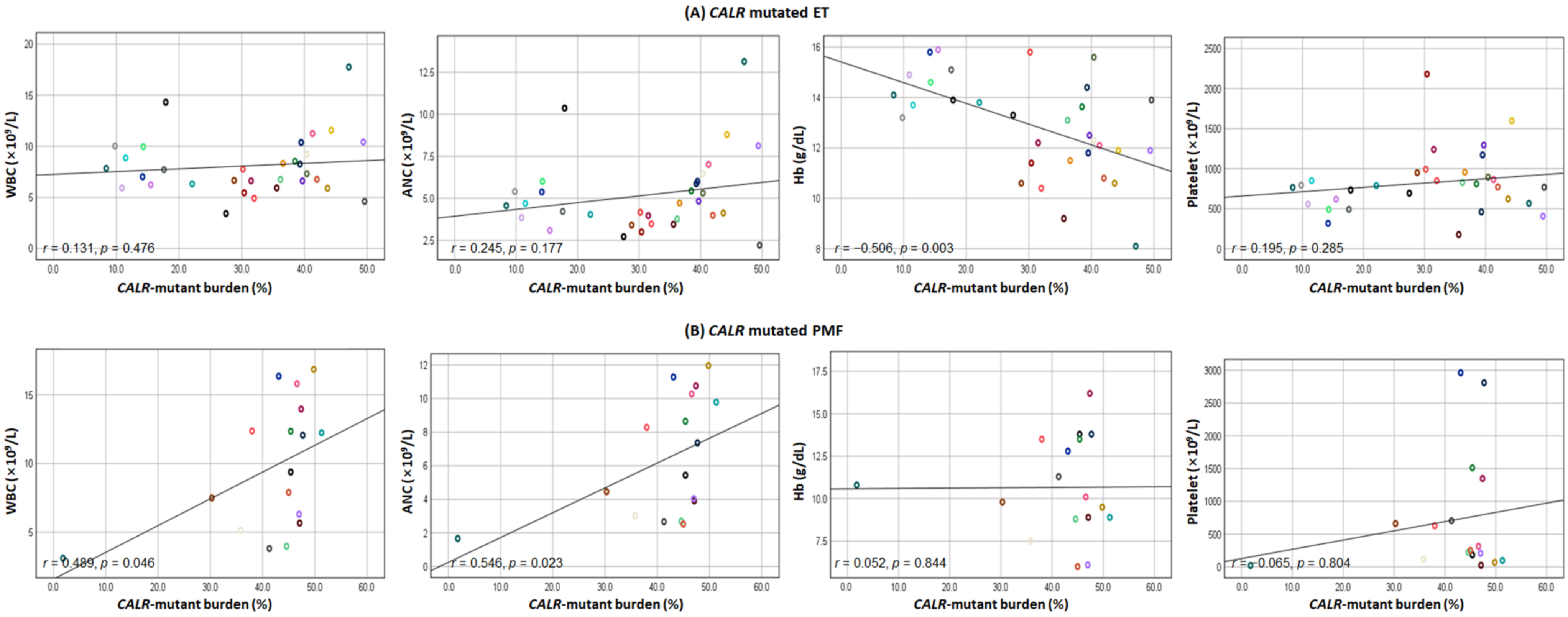

Supplement: Supplementary file 1 [file diagnostics-12-02570-s001.zip › diagnostics-1950580-supplementary.pdf]
